# Valorisation of food industry waste into high-performance biochar for environmental applications

**DOI:** 10.1038/s41598-025-11580-z

**Published:** 2025-07-19

**Authors:** Małgorzata Sieradzka, Wojciech Jerzak, Agata Mlonka-Mędrala, Anna Marszałek, Mariusz Dudziak, Izabela Kalemba-Rec, Aleksandra Błoniarz, Markus Reinmöller, Agnieszka Kopia, Wojciech Nowak, Aneta Magdziarz

**Affiliations:** 1 AGH University of Krakow, Al. Mickiewicza 30, 30-059 Krakow, Poland; 2 Silesian University of Technology, Konarskiego St. 18, 44-100 Gliwice, Poland; 3 Universität Stuttgart, Pfaffenwaldring 23, 70569 Stuttgart, Germany

**Keywords:** Food industry waste, Pyrolysis, Biochar activation, Porous materials, Pollutants adsorption, Environmental sciences, Engineering

## Abstract

Organic waste from the food industry can be thermochemically converted into valuable products, supporting the circular economy and reducing environmental and socio-economic impacts. This study explores the valorisation of food industry residues, such as rapeseed cake, maize cobs, and walnut shells, through slow pyrolysis at 600 °C under an inert atmosphere. The studied feedstocks were lignocellulosic materials with approximately 45% carbon content, but had different fibre contents, which subsequently affected the properties of the biochars. The highest char yield was observed for rapeseed cake (26%), followed by walnut shells (24%) and maize cobs (22%). Elemental analysis revealed that the carbon content in the biochars of maize cobs and walnut shells exceeded 80%, with a particularly significant surface area (356 m^2^/g) noted for the walnut shells, and very low for the rapeseed cake and maize cobs, respectively. Only after the chars underwent physical (steam activation at 850 °C) and chemical activation (using H_3_PO_4_ and ZnCl_2_), resulting in a substantial increase in surface area, exceeding 300 m^2^/g for rapeseed cake and maize cobs and c.a. 550 m^2^/g for walnut shell biochar. These biochars effectively removed organic (phenol) and inorganic (Pb^2+^) pollutants from aqueous solutions (100% removal of Pb^2+^ and 82% removal of phenol for biochar of walnut shells) and additionally did not exhibit acute toxicity in Lemna minor tests, confirming their environmental safety. The work aligns with SDG 6 (Clean Water and Sanitation) by enabling low-cost pollutant removal, SDG 12 (Responsible Consumption and Production) by upcycling food waste, SDG 13 (Climate Action) through carbon sequestration and emissions mitigation, and SDG 15 (Life on Land) by offering materials that improve soil health and support circular economy principles. This research demonstrates how engineered biochar can serve as a multipurpose environmental tool, directly supporting global sustainability targets.

**Supplementary Information:**

The online version contains supplementary material available at 10.1038/s41598-025-11580-z.

## Introduction

It is expected that global food demand will increase in a range of 59–98% by 2050^1^. An increase in food production will consequently lead to an increase in food industry waste. The generation of substantial quantities of organic waste represents a significant challenge for the effective management of waste and the broader goal of environmental sustainability. Waste can be converted through thermochemical processes into valuable products, supporting a circular economy (CE) and reducing the environmental, social, and economic burdens associated with waste disposal. The circular economy provides an alternative to the linear model, prioritizing resource preservation and emphasizing recovery and regeneration^2^. The use of residues from agriculture and food production as a feedstock for bio-based materials is a cost-effective, easily available, and abundant option. In contrast to agricultural waste, food industry by-products offer a more dependable and consistent supply, with a predictable composition over time, rendering them more suitable for large-scale conversion into carbon-rich materials^3^.

The biochar can be produced during thermochemical processes such as pyrolysis^4^. This product is distinguished by its high porosity^5^ and offers a multitude of applications, particularly in the domain of environmental remediation^6^. The efficiency of biochar in the capture CO_2_ and other pollutants has been well documented, as it offers a sustainable solution when addressing global environmental challenges, such as greenhouse gas emissions and water contamination^7^. The beneficial properties of biochar have been demonstrated in different studies^8,9^, including an improvement in water retention and a reduction in soil degradation. The alkaline nature and elevated nutrient levels of biochar derived from food waste contribute to enhancements in the chemical characteristics of soil^10^. Consequently, the incorporation of biochar into soil represents an effective approach to the improvement of soil quality and the remediation of contaminated soil^11^. The strategy to recover phosphorus for agricultural use represents a significant advance in the implementation of the previously mentioned circular economy concept.

The temperature commonly used for biochar production by pyrolysis is in the range of 400–700 °C^12–14^. These conditions minimize the production of pyrolytic oil and gas, while facilitating the production of biochar with an increase in the specific surface area of the biochar, thereby enhancing its adsorption properties for pollutants^15^. The pyrolysis process of food waste digestate residues for biochar production was studied by Wang et al.^16^, with particular focus on the pyrolytic properties, product formation, and behaviour of heavy metals. The research findings show that the digestate residue pyrolysis process consists of the following stages: moisture removal, decomposition of light volatiles; main pyrolysis associated with the decomposition of cellulose, hemicellulose, and proteins; decomposition of lignin and macromolecular substances; and decomposition of inorganic compounds.

Biochar produced at higher temperatures (around 700° C) exhibits improved characteristics, including higher surface area and reduced heavy metal toxicity. Recent developments have focused on optimising the pyrolysis process, which represents the primary method of converting food waste into biochar^17,18^. It is important to note that the high porosity and surface area of biochar are not the only factors that contribute to its suitability for the adsorption of organic and inorganic pollutants. The presence of various functional groups is also a determining factor in its effectiveness as an adsorbent. In addition, the effectiveness of biochar in the adsorption of heavy metals, including lead (Pb), cadmium (Cd) and chromium (Cr) has been documented. The adsorption mechanism involves the exchange of metal ions in solution with functional groups, such as hydroxyl (−OH) and carboxyl (−COOH), which are present on the biochar surface. This results in the formation of stable complexes^19^. As reported by Abdelhafez et al.^20^, Pb (II) removal capacity of biochar from orange peel was 28 mg/g, while the capacity of biochar from sugar cane was 87 mg/g. The adsorption capacity of cadmium (Cd^2+^), as presented by commercially activated carbon, was 2.5 mg/g. However, Ni et al.^21^ demonstrated that the use of biochar from anaerobically digested sludge can increase the capacity to almost 50 mg/g. Bone char samples were examined for uptake of Cr (VI), U (VI) and methylene blue adsorption, and it was found that the adsorption capacity was equal to 340 mg/g, 466 mg/g and 338 mg/g, respectively^22^. Furthermore, biochar has been identified as an effective solution for the adsorption of organic contaminants, including bisphenol A, with a removal rate of 3.2 mol BPA/(mol oxidant h)^23^. The high number of carbonyl groups on the surface of biochar allows for the removal of approximately 95% of phenol within 30 min during phenol ozonation^24^.

The production of biochar from food waste involves challenges related to feedstock variability, its influence on product properties, and the need for scalable, economically viable activation methods to enhance pollutant adsorption^25^. In this context, the present research addresses key aspects of sustainability and aligns with multiple Sustainable Development Goals (SDGs) through its innovative focus on waste valorisation and resource efficiency. By introducing novel biochar materials derived from food industry by-products, the study advances innovation in sustainable materials (SDG 9), promotes circular economy practices by repurposing waste into valuable products (SDG 12), tackles climate change through CO₂ adsorption capabilities (SDG 13), and improves soil quality through biochar valorisation (SDG 15).

This study explores the innovative use of by-products from the food industry, such as rapeseed cake, maize cobs, and walnut shells, to create high-performance biochar through pyrolysis followed by combined physical and chemical activation using H_3_PO_4_ and ZnCl₂. The research presents a novel approach to improving adsorption properties by employing customized activation strategies, supported by advanced characterization techniques. Additionally, it demonstrates the environmental significance of the resulting biochars through ecotoxicity testing and assessments of pollutant removal efficiency. The findings emphasize the untapped potential of underutilized food waste streams in producing sustainable materials that can be applied in environmental remediation and wastewater treatment.

## Materials and methods

### Materials

**Fig. 1:**
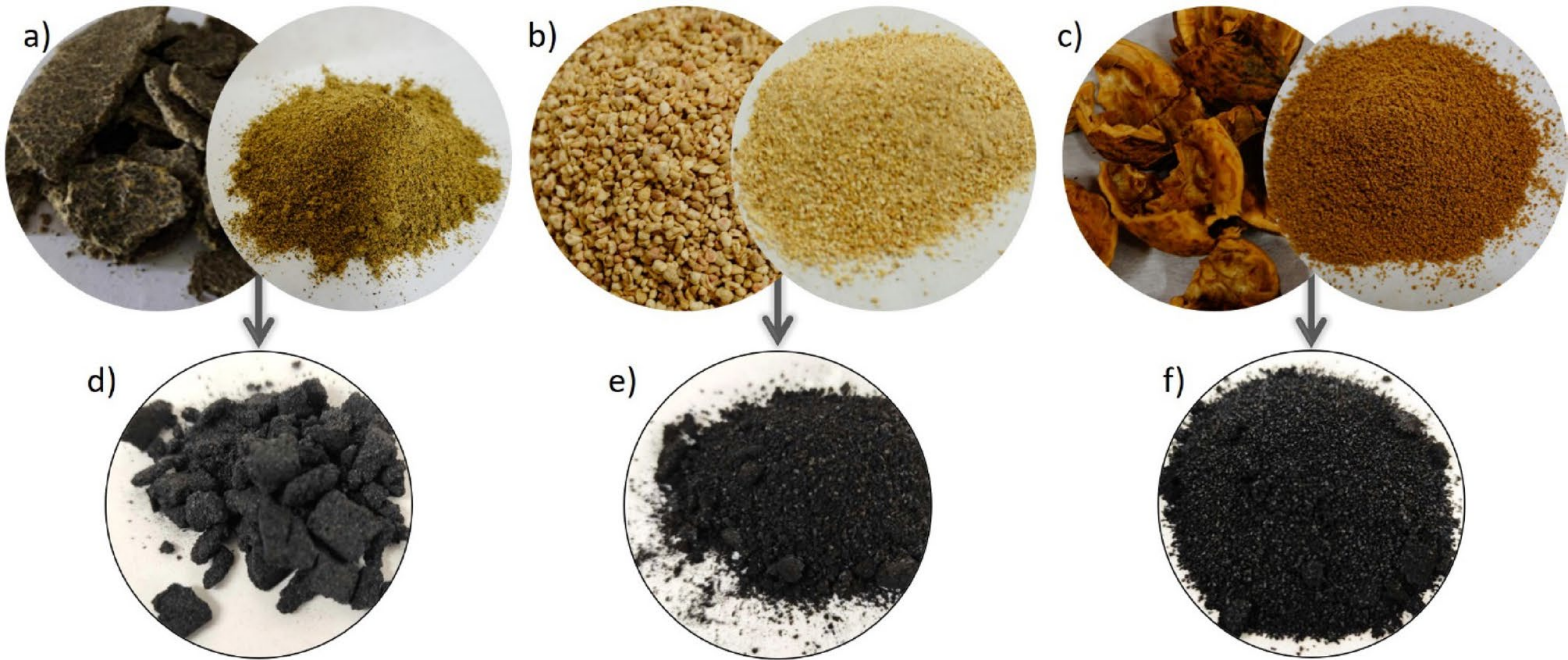
Images of studied feedstocks in raw form: (**a**) rapeseed cake (RC), (**b**) maize cobs (MC), and (**c**) walnut shells (WS), and biochar obtained under the pyrolysis process at 600 °C, and 10 min of residence time, (**d**) RC_char, (**e**) MC_char, (**f**) WS_char.

Biochars were produced from food wastes/residues such as rapeseed cake (RC), maize cobs (MC), and walnut shells (WS), and subsequently characterized in terms of their chemical composition, physical structure, and thermal behaviour. These properties, such as carbon content, porosity, and surface functionality of the resulting biochar, ensure their suitability for further applications. The studied biomass feedstocks included materials with significant potential due to their organic composition and renewable nature. The rapeseed cake is the solid residue left after the mechanical/pressure extraction of oil from the rapeseed, a maize cob is the central core of a maize fruit, and the walnut shells are the hard outer cover of the walnuts (Fig. 1). The studied feedstocks (raw biomass) were dried under ambient conditions for 24 h. Then the biomass was ground using a mill IKA MultiDrive control.

### Pyrolysis experimental procedure

The pyrolysis process was conducted in a horizontal fixed-bed furnace under a nitrogen (N_2_) atmosphere, with a flow rate of 100 mL/min. The reactor was heated to 600 °C, and the residence time was 10 min. The reactor was constructed from quartz glass, measuring 800 mm in length and 46 mm in external diameter, which allowed for the introduction of the sample into the heating zone after the experimental temperature. Once the sample was placed in the reactor, which was located outside the heating zone, the system was purged with nitrogen to eliminate any residual air. After purging, the sample was introduced into the heating zone. Following a 10-min period, the sample was removed from the heating zone and allowed to cool to ambient temperature in an N_2_ atmosphere.

### Analytical and Instrumental Methods

Proximate and ultimate analyses were performed to study the physical and chemical properties of the feedstock. Proximate analysis included the moisture content (*M*), volatile matter (*VM*), ash content (*A*), and fixed carbon (*FC*) as a solid combustible residue that remains after the volatile matter is released. The following standards were used to determine *M, VM* and *A*: EN ISO 18134–3:2023, EN ISO 18123:2023, EN ISO 18122:2022, respectively.

Ultimate analysis, which provided details concerning the elemental composition of carbon (C), hydrogen (H), nitrogen (N) contents, was determined using the Tuspec CHN628 LECO (St. Joseph, MI, USA) analyser according to the standard PKN–ISO/TS 12902:2007. The higher heating value (HHV) of feedstock was measured using a bomb calorimeter model LECO AC500 with respect to the standards DIN 51900 and ISO 1928.

Fibre analysis of the studied feedstock included determination of hemicellulose (HL), cellulose (CL) and lignin (L). The determination was based on analysis including aNDF (Neutral Detergent Fibre)—neutral detergent fibre, ADF (Acid Detergent Fibre)—acid detergent fibre, ADL (Acid Detergent Lignin)—acid detergent lignin. Contents of HL, CL and L were calculated based on the following formulas:

1 $$\text{HL}=\text{aNDF}-\text{ADF}$$

2 $$\text{CL}=\text{ADF}-\text{ADL}$$

3 $$\text{L}=\text{ADL}$$

The chemical composition of biomass ashes derived from the samples was determined using the X-ray Fluorescence method (XRF) with the WD-XRF spectrometer ZSX Primus II Rigaku (Rh irradiation source) (Rigaku, Japan). A qualitative analysis of the spectrum was performed by identifying the spectral lines and determining their possible coincidences and semi-quantitative analysis was developed using the SQX calculation program (fundamental parameter method).

The phase composition of ashes was analyzed using the X-ray Diffraction method (XRD). XRD patterns were acquired with a Panalytical Empyrean DY 1061 diffractometer (Malvern Panalytical, Almelo, The Netherlands) in Bragg–Brentano geometry with Cu K_α_ radiation (λ = 0.154 nm). A PDF-4+ database (ICDD, USA) was employed for phase identification.

The capacity of biochar to resist chemical and biological breakdown, referred to as recalcitrance, was determined using the thermogravimetric method. Thermal analysis was performed with a Mettler Toledo TGA/DSC Star System analyser (Mettler Toledo, Schwerzenbach, Switzerland). Analyses were performed under air (40 mL/min) in temperatures ranging from room temperature up to 900 °C in sapphire crucibles. Based on the method proposed by Harvey et al.^26^, the R_50_ index (Eq. 4) was used to assess the resistance of biochar to thermal oxidation during TG analysis, with graphite as a reference point. Graphite powder with an ultra-purity of 99.9995% (CAS: 7782-42-5) was purchased from Thermo Scientific Chemicals. Biochar and graphite had the same size fraction (100 mesh). Thermogravimetric curves were corrected for moisture and ash content.

4 $${R}_{50}=\frac{{T}_{50, biochar}}{{T}_{50, graphite}}\cdot 100\%$$

T_50,__biochar_ and T_50,__graphite_ are the temperature values corresponding to 50% mass loss due to oxidation of biochar and graphite, respectively.

The morphology and structure of the raw biomass and biochar were analyzed using Scanning Electron Microscopy (SEM) with an Inspect S50 instrument, coupled with Energy Dispersive Spectroscopy (EDS) (FEI Company, Oregon, USA) for elemental analysis. The images were obtained at an accelerating voltage of 10 kV in a low vacuum model.

Fourier-Transformed Infrared Spectroscopy (FTIR) (Bruker Corporation, Massachusetts, USA) was employed to analyze chemical groups of tested samples based on characteristic vibrations of chemical functional groups using Bruker Alpha II device conducting measurements in the range from 400 to 4000 cm^−1^.

The surface area of the samples was determined through nitrogen adsorption–desorption isotherm analysis and performed with a Micromeritics ASAP 2020 instrument (Micromeritics Instrument Corporation, Georgia, USA). The specific surface area (SSA) was calculated by applying the Brunauer–Emmett–Teller (BET) method. Total pore volume was determined from the amount of gas adsorbed at the highest relative pressure.

Wettability tests of raw biomass and biochar were performed using the DSA 25 KRÜSS goniometer (KRÜSS GmbH, Hamburg, Germany) with ADVANCE software. The contact angles (CA) with water were determined by the ellipse fitting method (sessile drop method). The fitting method used for CA determination is described elsewhere^27^. To ensure appropriate representativeness, a minimum of 10 measurements were taken for each sample, from which the average contact angle was then determined. To obtain a uniform surface of the material, the samples were affixed to a flat table using graphite tape. The contact angle was denoted within no longer than 2 s from the formation of a drop on the surface.

### Activation processes of biochar

Physical activation of the obtained biochar was conducted in a fixed-bed vertical reactor at 850 °C under a steam atmosphere, with nitrogen introduced as the carrier gas. First, the system was purged with nitrogen to eliminate any residual air. Next, the sample was heated from ambient temperature to 200 °C over 20 min to prevent steam condensation within the sample. Following this, the steam flow was initiated and the reactor temperature was increased from 200 to 850 °C, which was maintained for 15 min. After this period, the steam flow was stopped, and the sample was cooled in a nitrogen atmosphere until it reached ambient temperature.

A sequential chemical activation process using H_3_PO_4_ and ZnCl₂ was conducted to improve the specific surface area and microporosity of the resulting biochar. The use of H_3_PO_4_ helps preserve the porous structure of the material while also generating extensive porosity, resulting in diverse functional features on both the surface and within the bulk of the activated carbon. In contrast, activation with ZnCl₂ produces smaller pore sizes^28^. Based on Liang et al., activating with H_3_PO_4_ first, followed by ZnCl₂, led to the breakdown of P-O-C bonds and the formation of additional micropores^29^. Consequently, a similar activation approach was adopted in this study.

All experiments were performed in a horizontal fixed-bed reactor. Initially, phosphoric acid (H_3_PO_4_) with a concentration of 85 wt% was added to approximately 4.0 g of biochar in a 1:1 mass ratio (activator to biochar) to form a uniform paste. This mixture was left at room temperature for 12 h and then placed in an oven at 80 °C for 24 h. The prepared biochar-activator mixture was transferred to a crucible and loaded into the reactor. Prior to the experiment, nitrogen gas (100 mL/min) was used to purge the furnace for 15 min and continued to flow throughout the activation process. The first stage of activation was conducted at 350 °C for 1 h. After this step, this pre-activated sample was removed, washed with 3 mol/L HCl and deionized water until the filtrate reached a neutral pH, and then dried at 80 °C. Subsequently, the sample underwent a second activation step using ZnCl_2_. The activator was first dissolved in distilled water before being mixed with the pre-activated biochar in a 1:1 mass ratio. This second activation followed the same procedure as the first but was conducted at a higher temperature of 550 °C.

The biochars activated chemically and physically were named, e.g., WS_char_chem and WS_char_phys, respectively.

### Adsorption investigations

In order to determine the sorption capacity of biochar, the model water was prepared. A detailed description of the model water’s physicochemical characteristics can be found in literature^30^. The model waters contained inorganic contamination (lead concentration 300 μg/L, pH = 7.0) and organic contamination (phenol concentration 600 μg/L, pH = 7.0). Static adsorption studies were carried out on 100 ml glass bottles in a shaker at 320 rpm, of which the volume of each solution was 50 mL. The adsorbent concentration was 2 g/L and the adsorption time was 60 min. The initial concentrations of lead and phenol were selected to reflect environmentally relevant levels while ensuring detectable adsorption effects. The mass of the adsorbent and the solution volume were adjusted to maintain an appropriate solid-to-liquid ratio that provides effective contact between phases without oversaturating the adsorbent. All experiments were performed at ambient temperature (~ 22 °C). To establish adsorption isotherms under constant pollutant concentrations, different adsorbent dosages were employed: 0.4–2 g/L for lead and 1–5 g/L for phenol. This approach enabled the generation of a wide range of equilibrium concentrations (Ce) required for accurate isotherm modelling.

The experimental data were fitted by two common adsorption isotherms models: The Langmuir model, the Freundlich model. All calculations used in the article have been described in the previous paper by Marszałek et al.^31^.

The toxic effect was performed on aqueous extracts prepared on the basis of deionized water in accordance with the PN-EN 12457–4:2006 standard. A toxicity test was conducted using freshwater vascular plants *Lemna minor—Lemna sp*. Growth inhibition tests were based on observation of morphological changes in two-phase plants and were carried out at a temperature of 25 ± 1 °C with constant exposure to light with an illuminance of 6000 lx. Plants cultivated by the authors were used for the research. Both culture and toxicity tests were performed in accordance with OECD Guideline 221. Changes in plant morphology were observed after 7 days of exposure of the plant to the tested water extract and presented as a percentage of inhibition and growth of plant fronts according to Eq. (5), where L_k_ is the number of fronts for the control sample and L_t_ is the number of fronts for the test sample.

5 $$I=\frac{({L}_{k}-{L}_{t})}{{L}_{k}}\cdot 100$$

The interpretation of the results obtained was made in accordance with the toxicity classification listed in Table S1 (see Supplementary materials).

## Results

### Material characteristics

The results of the proximate, ultimate analysis and HHV are presented in Table 1. All values are presented as means ± standard deviation (n = 3). Proximate analysis reveals similarities between each of the studied samples, with volatile matter content ranging from 76.93 to 80.37%, whereas the ash content ranged from a minimum of 1.28% for walnut shells (WS) to a maximum of 6.94% for rapeseed cake (RC). From a thermochemical process perspective, a high VM content and relatively low ash content are preferable, as it allows for the production of char with a higher organic content and a complex porous structure due to the release of gaseous compounds under high temperatures^32^. From the other side, as demonstrated by Chun et al.^33^, feedstocks characterised by higher ash content resulted in reduced char yields following pyrolysis at 500 °C Ultimate analysis presents carbon and hydrogen contents in the range of 44.46% to 48.49% and 6.31% to 7.09%, respectively, which are in line with the composition of other food waste samples^34,35^. The high nitrogen content in the RC can be attributed to the significant quantity of proteins in the sample and is indicated by the origin of this feedstock^36^.

**Table 1:** Proximate, ultimate and higher heating value analysis of the studied biomass waste.

Parameter	RC	MC	WS
Moisture content (M), wt%	6.76 ± 0.06	5.75 ± 0.12	5.44 ± 0.24
Volatile matter (VM) wt%	76.93 ± 0.17	80.37 ± 0.02	79.59 ± 0.48
Ash content (A), wt%	6.94 ± 0.08	1.86 ± 0.31	1.28 ± 0.09
Fixed carbon (FC), wt%	9.37 ± 0.23	12.02 ± 0.17	13.69 ± 0.33
Carbon (C), wt%	45.86 ± 0.06	44.46 ± 0.08	48.49 ± 0.07
Hydrogen (H), wt%	7.09 ± 0.02	6.31 ± 0.06	6.38 ± 0.07
Nitrogen (N), wt%	4.86 ± 0.05	0.30 ± 0.04	0.30 ± 0.00
Oxygen (O), wt. %	35.25 ± 0.08	47.08 ± 0.11	43.55 ± 0.14
HHV, MJ/kg	19.79 ± 0.01	17.3 ± 0.08	19.22 ± 0.05

The studied food waste was characterized by its different fibre composition which had, as a consequence, the properties of received biochar, e.g. thermal stability, surface area and adsorption capacity (see Table S2, supplementary materials). The maize cobs (MC) waste reveals the highest cellulose content (45.32%), followed by RC (34.31%). Cellulose is distinguished by its high degree of crystallinity and strong hydrogen bonding capabilities, attributable to the hydroxyl groups present in its structure. These features enhance its structural stability but can limit its adsorption capacity by reducing available active sites. Glycosidic bonds in cellulose requires lower temperatures and residence times than lignin^37^, resulting in almost complete thermal decomposition of this fibre during pyrolysis process (see Table 2). However, it has been demonstrated that modified or fractionated cellulose can effectively adsorb contaminants such as heavy metals and dyes by providing a structured and relatively porous matrix. Hemicellulose, a branched polysaccharide (4.16% in RC to 10.80% in MC), exhibits an amorphous structure, which allows for easier chemical modification and interaction with pollutants.

**Table 2:** Yield, fibre and elemental analyses of biochar obtained from the pyrolysis of studied biomass waste.

Parameter	RC_char	MC_char	WS_char
Yield, wt. %	25.85 ± 0.16	22.31 ± 0.38	24.08 ± 0.16
Hemicellulose (HL), wt%	2.04 ± 0.40	4.98 ± 0.73	3.27 ± 1.43
Cellulose (CL), wt%	0.87 ± 0.33	2.30 ± 0.10	1.18 ± 0.93
Lignin (L), wt%	34.90 ± 2.41	40.96 ± 10.38	36.82 ± 0.89
Carbon (C), wt%	61.85 ± 1.18	84.07 ± 1.71	87.04 ± 0.35
Hydrogen (H), wt%	2.41 ± 0.19	2.71 ± 0.12	2.74 ± 0.16
Nitrogen (N), wt%	5.49 ± 0.10	0.65 ± 0.04	0.72 ± 0.01

It has been demonstrated that it is capable of effectively adsorbing certain metal ions; however, its lower stability in comparison to cellulose and lignin has the potential to limit its long-term effectiveness^38^. Lignin, which ranges from 14.18% in RC to up to 37.57% in WS, is highly aromatic and contributes significantly to the structural integrity of biomass. Research indicates that lignin exhibits a high affinity for organic contaminants and dyes due to its complex structure and availability of functional groups such as hydroxyl and methoxy moieties^38^. The presence of lignin has been shown to enhance the material’s resistance to degradation, which can be advantageous for applications involving long-term adsorption^38^. Lignin pyrolysis requires a larger temperature range than cellulose and hemicellulose, it was observed that higher yield of char can be obtained through thermal conversion of lignin-rich materials^37^. Finally, it can be concluded that rapeseed cake, which has moderate cellulose and a low lignin content, can be easily degraded for use as animal feed or for bio-conversion. Maize cobs, with their high cellulose content, are suitable for bioethanol production. In contrast, walnut shells, which have a high lignin and a very low cellulose content, are more appropriate for energy generation or the production of carbon-based materials rather than for biofuel conversion.

The analysis of the chemical composition of ash, based on results from the X-ray fluorescence spectrometer (see Table S3, Supplementary materials), indicated significant differences in the distribution of major elements among the studied materials. Furthermore, the chemical composition of biomass waste ash provides valuable insights into its potential impact on biochar applications as a catalyst or adsorbent, as evidenced by this research. As demonstrated in previous studies^39^, potassium (K) and calcium (Ca) are primary identified elements in biomass ash. However, the specific composition of these elements varies depending on the type of biomass source and other influencing factors^40^. The presented results illustrate a consistent correlation for the WS sample, with K_2_O equal to 51.72% and CaO 25.57%. Additionally, the presence of potassium may be advantageous for utilization in fertiliser applications^8^. In the case of MC, potassium was the primary ash component (71.50%), while in RC, it was the secondary main compound (25.08%). For RC, the first component was P_2_O_5_ (39.86%), suggesting that this material is rich in phosphorus-containing compounds, likely due to its biological composition, which makes it a material with potential for phosphorous recovery. The mineral fractions contained within ashes have been shown to exert an effect on the sorption properties of Cd, according to the findings of studies conducted on the subject. It is well established that mineral-rich biocarbon is typically alkaline in nature and has the capacity for high and strong metal sorption^41^.

Phase composition analysis has confirmed the presence of various potassium, calcium, and phosphate compounds. The predominant phase of RC was identified as K_2_CaP_2_O_7_, whereas, the WS sample exhibited fairchildite K_2_Ca(CO_3_)_2_, and the MC sample displayed kalsilite (KHCO_3_). The KHCO_3_ phase has the potential to reversibly transform to K_2_CO_3_, within a temperature range of 100 °C and 200 °C^8^.

The results of the yield, elemental and fibre analysis of the biochar obtained after pyrolysis are presented in Table 2. Pyrolysis resulted in similar biochar yields (22–26 wt%) across the various raw materials studied. The obtained pyrolysis yields at 600 °C align well with previously reported data, including yields of 23% for rapeseed cake^42^, 26% for corn cobs^43^, and 21–24% for walnut shells^44^. Physical activation showed higher yield in comparison to pyrolysis process (70–81%) across all studied materials. The yield of biochar following steam activation can demonstrate variability. For instance, a study documented a mass yield of 88 wt% for steam-activated biochar^45^. A further study^46^ successfully attained a maximum biochar yield of 42.3 wt% through the utilisation of a dual fluidised bed system, employing a combination of steam and phosphoric acid. As it was predicted^41^, the pyrolysis process resulted in the decomposition of the fibres contained within the raw materials. This phenomenon was most evident in the cases of hemicellulose and cellulose, where a decrease of approximately 50% and 95%, respectively, was observed in the original values of these compounds. The decrease in cellulose was particularly pronounced in RC and MS, with a loss exceeding 95%, while in WS it was over 70%. The increase in lignin content in the sample is attributable to the higher decomposition temperature of this compound in comparison to the others. As cellulose and hemicellulose undergo greater decomposition than lignin, the lignin content of the carbonate increases, although a proportion of its structure also decomposes. Furthermore, as indicated in the study^47^, there are significant interactions between cellulose, hemicellulose and lignin during the pyrolysis process. The presence of hemicellulose, which was the highest in both RC and MC, led to the disappearance of the exothermic peak of lignin, resulting in a decrease in the degradation of the compound. It was observed in Liu et al. study^48^ hemicellulose promotes the pyrolysis of lignin by shifting it to a lower temperature range and significantly reducing the mass loss rate. As revealed by Windeatt et al.^49^, there is a link between carbon content in biochar and lignocellulose content in raw material. A higher lignin content provides a higher carbon density char, while cellulose content decreases the carbon content in char. The highest carbon values were observed in the MC_char and WS_char samples, which also had the highest lignin content of 41% and 37% for MC and WS, respectively. Despite the RC sample containing 46% carbon, its biochar contained only 62% of this element, due to its high cellulose content (35%). Although MC exhibited a higher cellulose content (45%) compared to RC, the carbon content of biochar was found to be higher due to its greater lignin content.

### Structural and morphological analysis

**Fig. 2:**
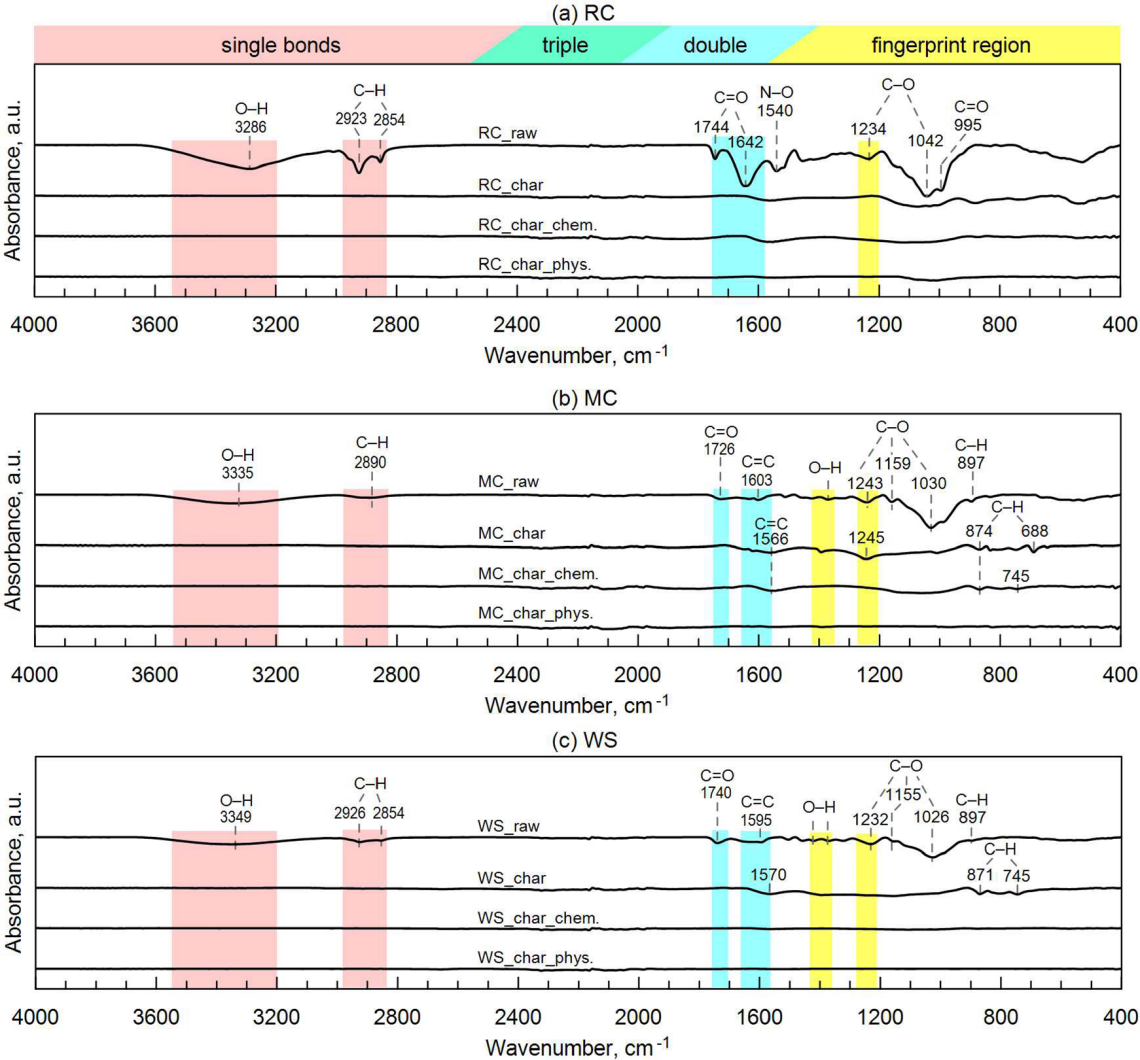
FTIR spectra of feedstocks and biochar: (**a**) rapeseed cake, (**b**) maize cobs, and (**c**) walnut shells.

The Fourier-Transform Infrared Spectroscopy spectra of the raw materials and obtained biochar are presented in Fig. 2. The absorption peaks observed at 688 cm^−1^ and 745 cm^−1^ wavenumbers corresponds to the stretching vibration aromatic bond of C–H^50^. This bond was observed for WS_char and MC_char, as well as for MC_char_chem. Peaks assigned to C=O and O–H bonds appeared only in the raw materials. Their absence in the biochars indicates that decarboxylation and decarbonylation reactions occurred during pyrolysis, resulting in a weakened electron-donating ability of the biochar^51^. Aliphatic compounds were relatively stable in raw materials, which is indicated by peaks between 2850–2926 cm^−1^, which corresponds to the C–H stretching vibration^52^. The evolution of major surface chemical functional groups, including phenolic O–H (3350–3280 cm^−1^), aliphatic –CH_n_ (2930–2854 cm^−1^), aromatic rings of C=C double bonds (1603–1370 cm^−1^), and aromatic C–O (1245–1020 cm^−1^) diminished or stabilized in biochar (confirmed by^53^). This finding suggests that reactions of depolymerization and decomposition occurred, which resulted in the detachment of side chains and bridge bonds of aliphatic structure from the raw material organic matrix^53^.

**Fig. 3:**
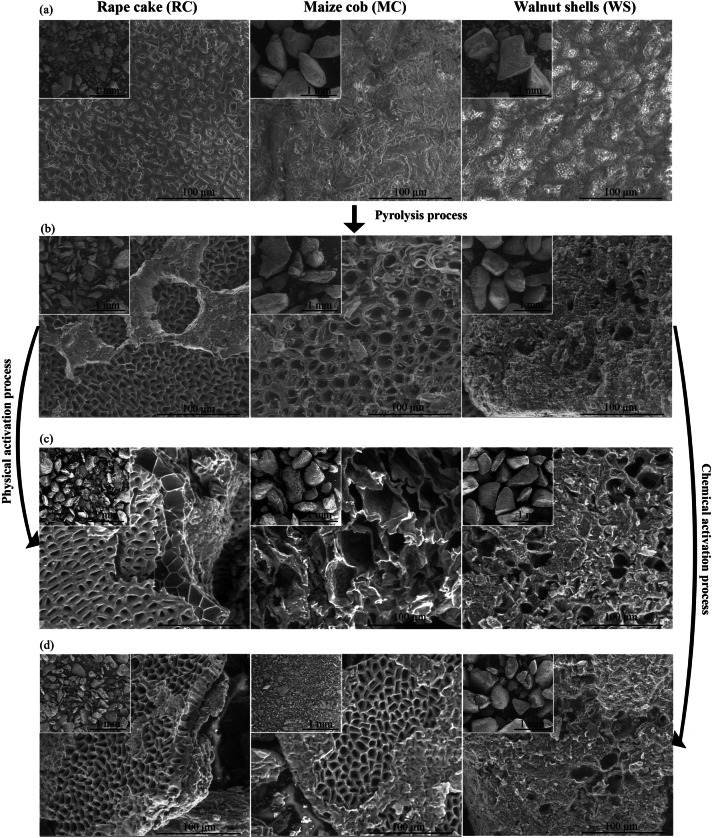
SEM image of (**a**) raw biomass, (**b**) biochar, (**c**) physically activated biochar, (**d**) chemically activated biochar.

Figure 3 presents microphographs of the biomass, biochar, and biochar after physical and chemical activation processes. Morphology of the analyzed biomass samples were varied. The particles of rapeseed cake were finer in comparison to particles of maize cobs and walnut shells. Some particles of maize cobs and walnut shells were larger than 1 mm in size. The surface of rapeseed cake particles was smooth with shallow hollows. Maize cob and walnut shell particles were characterized by a rougher surface. In the case of walnut shells, small holes were visible on the surface. The pyrolysis process resulted in the development of an internal porous structure, as seen in the SEM images of the biochar (Fig. 3b). The biochar of walnut shells had the lowest porosity. After the activation processes (Fig. 3c, d), the porosity of the biochar particles increased, especially after physical activation. Although the physical activation process did not affect the particle size of biochar. However, after the chemical activation process, the maize cob particles were significantly fragmented. Among the tested biochar, only particles of walnut shells were slightly damaged in their structure. After chemical activation, all examined biochar contained additional zinc and chlorine, as indicated by the EDS analysis (see supplementary materials, Fig. 1S).

### Thermal stability of biochar

**Fig. 4:**
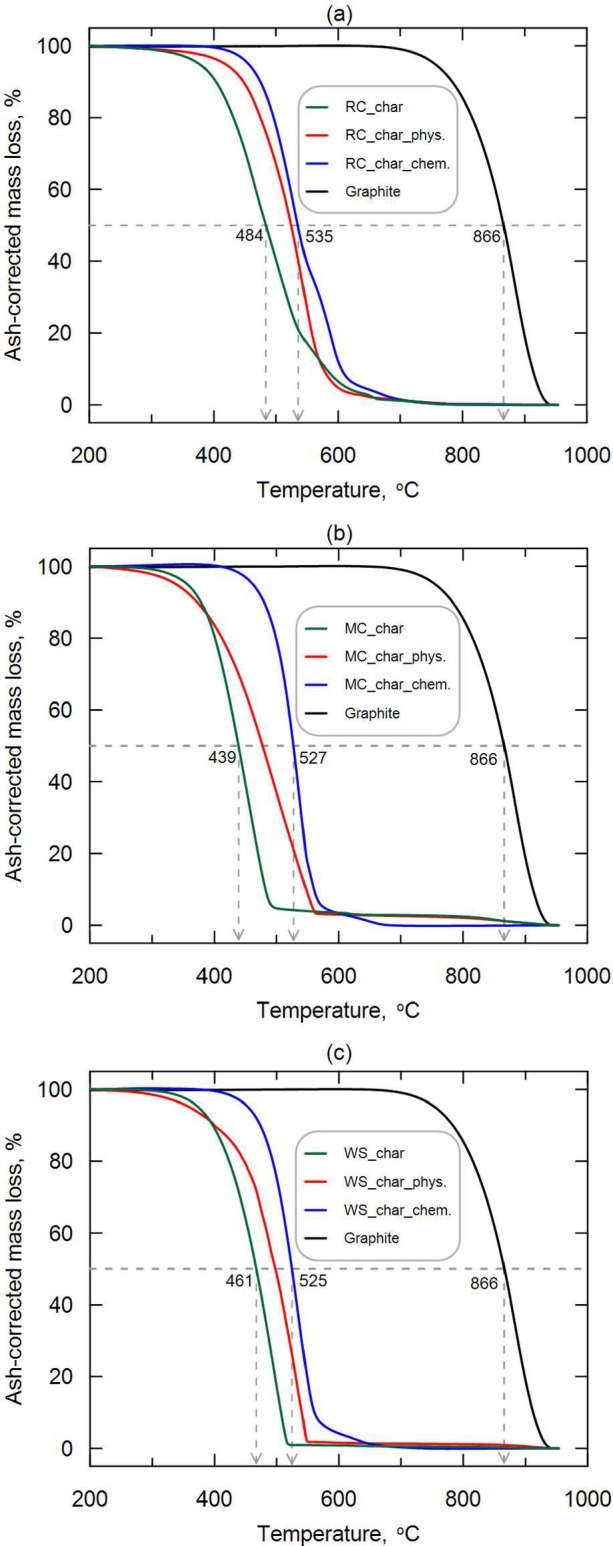
Corrected thermogravimetry curves of biochar (char) from (**a**) rapeseed cake, (**b**) maize cobs, and (**c**) walnut shells, and biochar activated physically (char_phys) and chemically (char_chem) compared to graphite.

Thermogravimetric curves corrected for moisture and ash content of biochar are presented in Fig. 4a-c. The thermal degradation of biochar occurs in three distinct stages. The first stage, between 200 and 400 °C, shows minimal mass loss (< 20%) for nonactivated char and char_phys, while char_chem remains stable with no measurable loss. The second stage, which begins above 400 °C, is marked by significant mass loss, reaching its peak between 500 and 600 °C, depending on the type of biomass and activation method. This rapid degradation is driven by the low-temperature oxidation of char. The specific temperatures at which maximum mass loss occurs are provided in Fig. 2S (see Supplementary materials). In the final stage, above 600 °C, no further mass loss is observed.

As shown in Fig. 4a–c), the following T_50_ trends were observed for each biochar: char < char_phys < char_chem. This confirms that the activation of biochar promotes an improvement in environmental resistance. The temperatures at which 50% biochar weight loss occurred ranged between 439 °C (MC_char) to 535 °C (RC_char_chem). At 866 °C there was a 50% weight loss of graphite. A slightly higher value of T_50_ = 886 °C for graphite was found when a four times lower air flow rate was used compared to the presented studies (40 mL/min)^26^. Thermal stability of biochar was studied among others by Zhao et al.^54^, who reported that 50% mass loss occurred at temperatures from 467–710 °C. At the same time, it was shown that the higher the pyrolysis temperature of biochar, the higher the temperature at which 50% mass loss occurred.

**Fig. 5:**
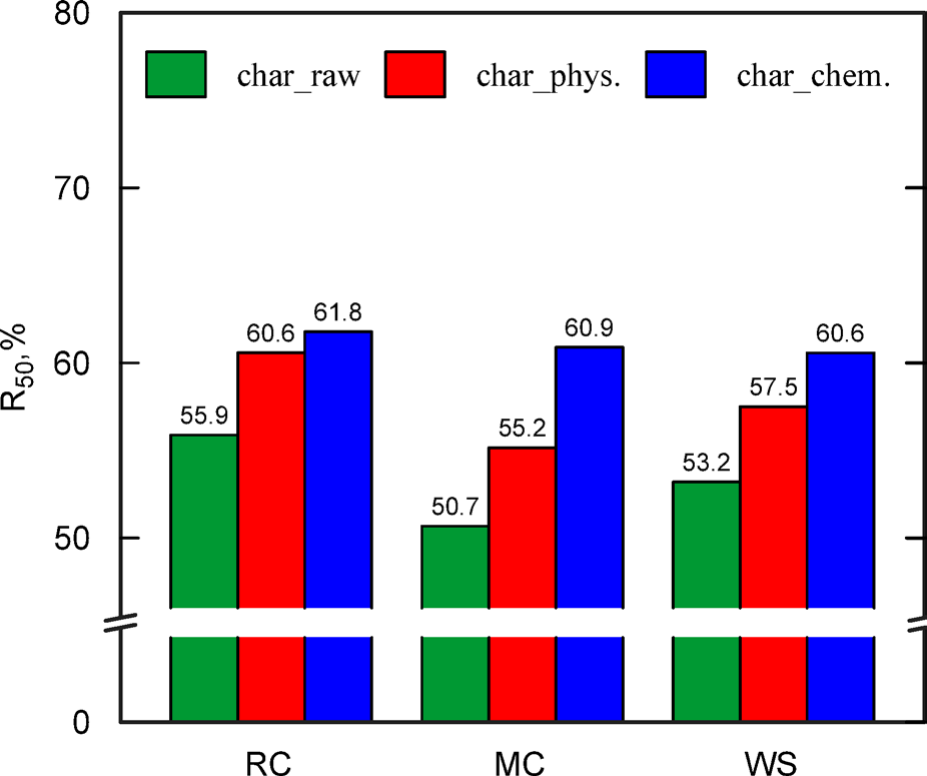
Stability of biochar in terms of resistance to thermal-oxidation based on thermogravimetric analysis.

Figure 5 shows the calculated R_50_ indexes from Eq. (4), using the T_50_ values (illustrated in Fig. 4a–c). Based on the R_50_ resistance classification system^26^, all types of biochar are in class B (50% < R50 < 70%), i.e. slightly biodegradable. The highest R_50_ values of biochar were obtained for RC_char_chem < MC_char_chem < WS_char_chem. This confirms that the chemical activation of biochar is more effective in increasing its oxidative stability (i.e., reducing biodegradability) compared to physical activation. When comparing nonactivated biochar to physically activated biochar, an increase in R_50_ values of 4.3–4.8% was noted. There is no apparent correlation of lignin content in biomass on the R_50_ value, as reported in other papers^49^.

**Fig. 6:**
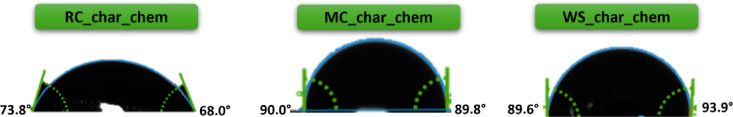
Image of a liquid droplet on the surface of chemically activated biochar.

The wettability of the investigated sample types was assessed by the sessile drop method, which determines the contact angle between water drop and biochar surface. Regarding biomass before the pyrolysis process (RC, MC, WS) and the appropriately obtained biochar (RC_char, MC_char, WS_char), including physically activated biochar, the rate of water droplet penetration into the sample was less than 1 s. This resulted in the inability to accurately determine the value of the contact angle (CA). However, considering the time of droplet penetration into the sample, it can be concluded that these materials were hydrophilic^55^. The hydrophilic properties of the above-mentioned samples may result from the temperature conditions of the pyrolysis process (600 °C)^55,56^. It has been demonstrated that different types of biochar produced at temperatures < 500 °C are hydrophobic, while those produced at a higher temperature (> 500 °C) tend to increase hydrophilicity. This is related to the effect of releasing pores clogged with tar, due to aliphatic compounds evaporating at a high temperature. Furthermore, this confirms the thesis that all the investigated samples, except for biochar after chemical activation, indicate a hydrophilic character. Figure 6 presents the image of an example droplet obtained in the first second of measurement of these materials. The contact angles measured for chemically activated biochars, specifically MC_char_chem (92.2° ± 5.3°) and WS_char_chem (93.2° ± 5.8°), exceed 90°, which classifies them as hydrophobic materials^57^. This surface property is particularly relevant in environmental applications such as wastewater treatment, where hydrophobicity can enhance the adsorption of non-polar organic pollutants and improve separation efficiency in multiphase systems. RC_char_chem, with an average contact angle of 78.4° ± 11.5°, is a moderately hydrophilic material. However, it should be emphasized that by virtue of its significant heterogeneity, depending on the measurement location, CA values above 90° were obtained for RC_char_chem, as indicated by the significant standard deviation error. It should also be taken into account that all standard deviations from the average are considerable which is a result of the complexity of the materials and, thus, the problematic samples influence on measurements. Depending on the chemical treatment used, the properties of biochar can be selectively changed by controlling the functional groups, which results in greater application possibilities^58^.

Chemical activation and other methods allow for changes to the surface properties of the material. For example, Li et al.^59^ obtained a highly hydrophobic catalyst, excellent for use in biofuel production, by chemically activating biochar obtained from coconut shells with benzosulfinic acid. Zhang et al.^60^ chemically modified biochar obtained from rice straw in the pyrolysis process (500 °C). The application of the silane coupling agent KH-579 improved the hydrophobicity of the biochar, which may allow for its effective use as an additive to the soil cover in landfills This would provide it with greater water resistance. In our studies, chemical activation also increased the hydrophobicity of the obtained materials, as evidenced by the fact that when determining the exact value of the CA, only chemically modified biochar was viable in the tests. In addition, MC_char_chem and WS_char_chem are materials classified as hydrophobic based on the CA value above 90°.

### Specific surface area and pore distribution

Rapeseed cake, after the pyrolysis process, displays properties similar to raw feedstock with a negligible surface area (Table 3). This could be associated with the lowest (among analysed samples) content of hemicellulose and cellulose in the sample, as the porous structure during the pyrolysis process is developed when volatile substances, produced by the decomposition of lignocellulose in the biochar, escape and pores are formed^61^.

**Table 3:** Surface area and pore structure of the studied biochar (where: BET—Brunauer, Emmett and Teller, BJH -Barrett-Joyner-Halenda).

Studied material	Specific surface area (BET), m^2^/g	Micropore Vol., cm^3^/g	Mean pore diameter (adsorp. BJH), nm	Mean pore diameter (desorp. BJH), nm
RC_char	0.25	–	–	–
RC_char_phys	302.8	0.1027	6.05	3.86
RC_char_chem	441.18	0.1466	9.06	5.07
MC_char	6.18	0.0023	48.52	–
MC_char_phys	315.72	0.1150	5.58	2.65
MC_char_chem	537.59	0.1873	4.33	2.69
WS_char	355.92	0.1243	4.71	2.87
WS_char_phys	544.96	0.1813	5.02	3.51
WS_char_chem	557.09	0.1922	4.37	2.71

After activation using steam gasification, both the surface area and the micropore volume increased. The data suggests the physical treatment opens up further smaller pores, with some larger pores appearing in the desorption phase. Chemical treatment resulted in a larger increase in the surface area and the material overall had a better-developed micropore structure. Compared to physical treatment, chemical treatment has both smaller and larger pores and seems to favour mesopore formation (larger pores).

The maize cob pyrolysis process developed a very low specific surface area in the obtained char, which suggests that this material might have a macroporous structure. Further treatment using steam activation resulted in a substantial increase in surface area and micropore volume. However, the mean pore diameter is slightly larger than the raw char, indicating a shift towards mesoporosity. It was observed in a study performed on tea waste that despite the fact that the microporous volume increased, it does not directly indicate the development of a microporous structure. Furthermore, as the micropore volume fraction decreased, the steam activation temperature increased, and micropores widened, resulting in mesopores^62^. The chemical treatment of MC_char resulted in the highest surface area and micropore volume. The material has mostly small to medium pores (micropores and mesopores), suggesting that the chemical treatment optimises the pore structure for both adsorption and desorption.

Walnut shell-derived biochar has a moderate surface area and micropore volume, showing potential as an adsorbent, with mesopores appearing during both adsorption and desorption phases. Physical treatment of biochar greatly increases the surface area and micropore volume, enhancing its adsorption properties. The material has small pores during adsorption but larger pores appear after desorption, which might be a result of the partial clogging of pores with tar. The chemically treated walnut shell char indicates a slightly higher increase in surface area and micropore volume. Similar to other chemically treated materials, it favours small to medium-sized pores, ideal for adsorptive purposes. Satisfactory results regarding physical activation with only a small improvement using chemical methods was also noted regarding the example of coconut shells in Prauchner et al., which might have similar properties to walnut shells^63^.

Chemical treatments consistently provide the highest surface areas (e.g., MC_char_chem, WS_char_chem), which is key for applications such as adsorption. Micropore volume was maximised in the chemically treated samples, indicating that chemical treatments create more microporous structures, which is important in adsorption processes. Additionally, chemical treatments result in the smallest pores (based on BET adsorption results). BJH analysis indicates that chemical treatments also produce more mesoporous structures (medium-sized pores), which may be beneficial for adsorptive processes that require both micro- and mesoporous structures.

Based on previous research on food waste, for example, fruit waste, it was concluded that temperature is one of the most important parameters affecting the porous structure of the material. Moreover, the introduction of steam at high temperatures helps eliminate volatile substances produced during pyrolysis, which causes the active sites on the biochar surface to open up and new pores to form within the structure^64^. According to Qin et al. conditions for steam activation were found to be around 850 °C, but the extension of residence time up to 60 min may result in better results^65^. Steam activation also promotes the creation of new micropores and widen existing ones, although excessive activation could decrease the surface area and total pore volume^66^.

Residence time is the second most important parameter in physical activation. Based on the analyses of mixed food waste, it was observed that a shorter residence time favours specific surface area development^67^.

In the presented study, it was reported that the type of food waste and especially its fibre content directly affects the potential of the material towards the synthesis of porous carbon materials of a high specific surface area and microporous structure. Based on the fibre content, a proper synthesis method should be selected, taking into consideration other environmental factors such as energy consumption and waste generation. While physical activation is a cost-effective process with a lower environmental impact, chemical activation is often preferred due to its ability to enhance porosity^68^. However, as demonstrated in this study, in some cases both methods provide similar results.

### Adsorption of pollutants

**Fig. 7:**
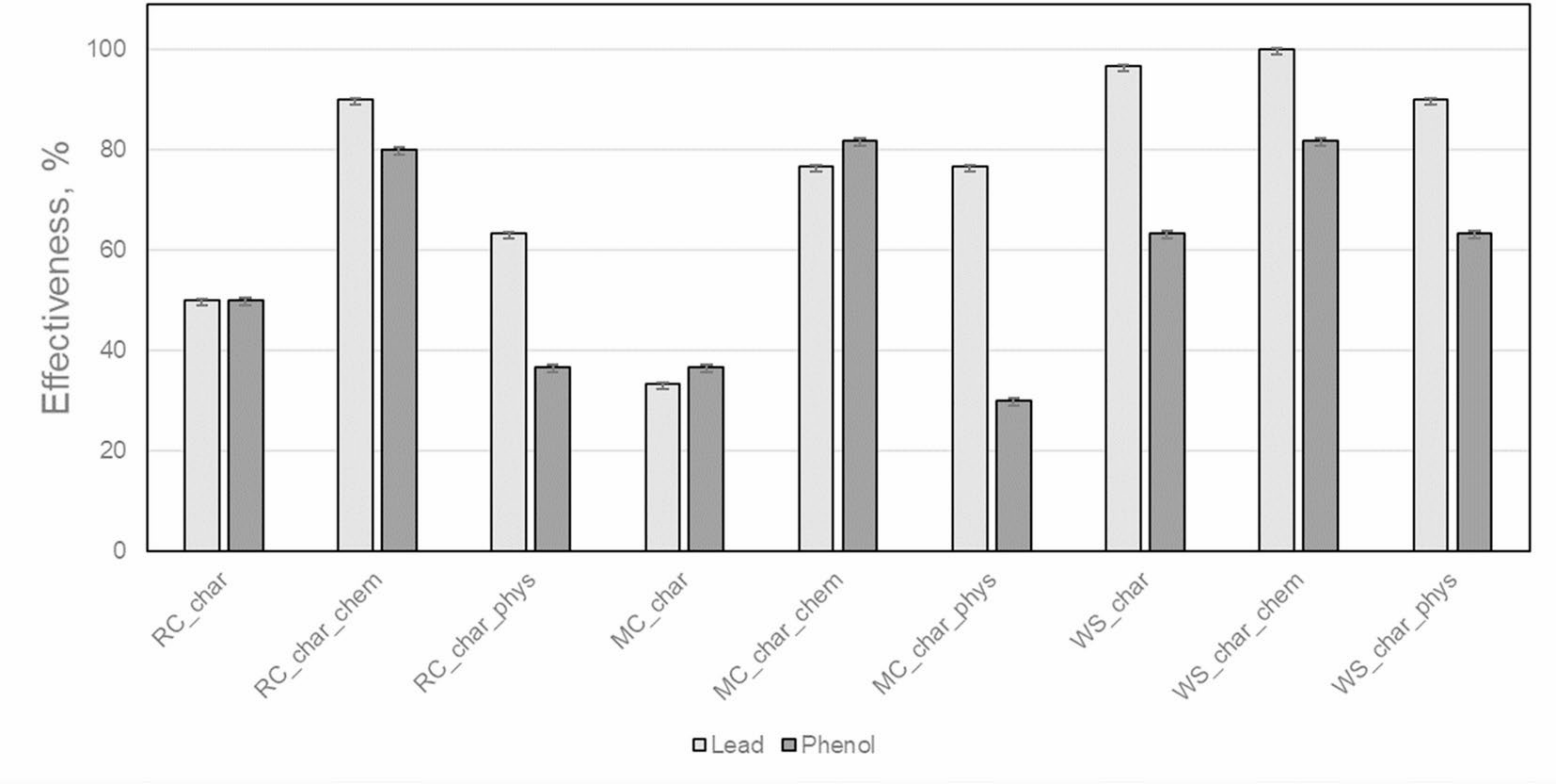
Adsorption efficiency of lead and phenol from the studied biochar.

The adsorption efficiency of lead and phenol from deionised water for the adopted adsorption conditions is shown in Fig. 7.

On the basis of the research carried out, it was found that all of the biochar had showed sorption properties against both organic and inorganic pollutants. Among the studied nonactivated biochar samples, the highest adsorption efficiency of lead and phenol was demonstrated by WS_char biochar (97% for Pb and 63% for phenol). Biochar of rapeseed cake (RC_char) and maize cobs (MC_char) were characterized by their lower adsorption efficiency. Chemical activation significantly increased the adsorption efficiency for all studied biochar types. In the case of WS_char_chem, the highest adsorption efficiency was achieved for both lead (100%) and phenol (82%). Ahmad et al.^69^, in a review on the use of biochar, confirm that chemical activation significantly improves the sorption capacity of biochar, which was also confirmed by this investigation. It indicates that chemical activation increases the number of functional groups, improving the sorption capacity. Additionally, it can be emphasized that the effectiveness of biochar in the adsorption of various organic/inorganic pollutants is still uncertain and requires further investigation. Physical activation also improved the sorption capacity, but its effectiveness was lower than that of chemical activation. Phytotoxicity tests conducted using Lemna minor showed that in all studied samples, plant growth inhibition was less than 25% and according to the ISO 20079 standard, classifies them as non-toxic. This result suggests that none of the tested biochars pose an acute risk to aquatic organisms, including even the chemically activated samples. This is consistent with broader studies indicating that many biochars, especially those produced at higher pyrolysis temperatures or from certain feedstocks, generally exhibit low acute toxicity to aquatic organisms, including algae and invertebrates^70,71^.

The literature reports the potential toxicity of some biochars resulting from the presence of organic contaminants such as polycyclic aromatic hydrocarbons (PAHs) or heavy metals. For example, studies conducted by Gale et al. (2016) indicate that some biochars may release toxic substances that negatively affect aquatic organisms^60^. Other studies have found that biochars can release water-soluble organic compounds, free radicals, or residual heavy metals that can cause toxicity to aquatic microorganisms and algae, particularly when produced at lower temperatures or from contaminated feedstocks^72,73^. In this study results indicate that the appropriate selection of feedstock and modification method, especially chemical activation, can minimise this risk. Environmental factors such as pH, ageing, and the presence of natural organic matter can also further influence biochar toxicity, sometimes minimising or, in other cases enhancing adverse effects^74^. Additionally, the low toxicity recorded even for WS_char_chem indicates the effectiveness of post-activation purification procedures and the absence of release of undesirable substances that could impact the aquatic environment^75^. Therefore, it was confirmed that biochar, particularly walnut shell biochar with chemical activation, provides a high adsorption efficiency and is safe for aquatic environments. This supports potential application in water treatment without ecological risks.

**Fig. 8:**
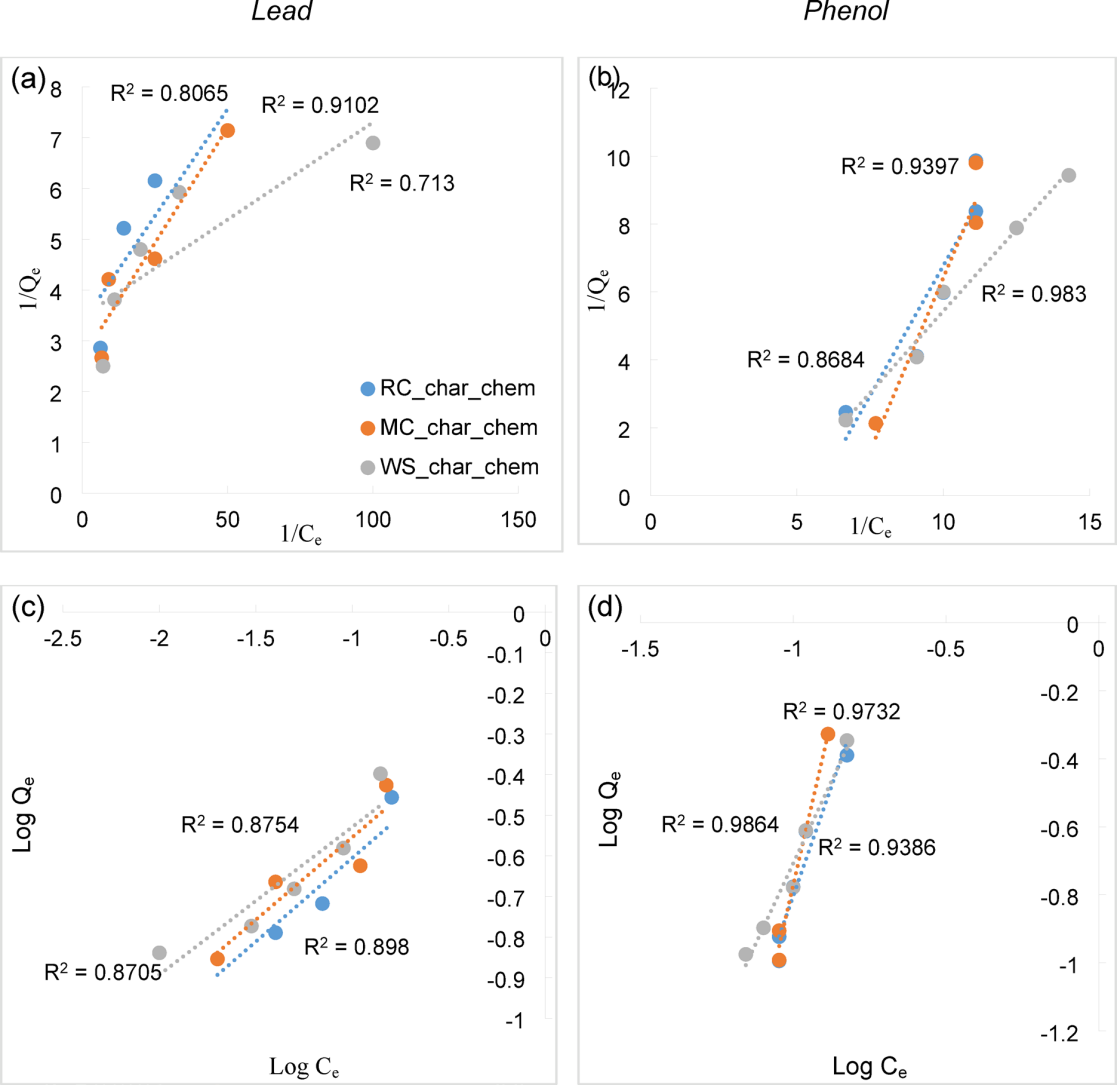
Langmuir (**a**), (**b**) and Freundlich (**c**), (**d**) adsorption isotherms for lead and phenol on RC_char_chem, MC_char_chem, and WS_char_chem biochars.

Due to the significantly higher sorption efficiency observed for chemically activated biochars, detailed modelling of adsorption isotherms Langmuir and Freundlich was performed only for RC_char_chem, MC_char_chem and WS_char_chem samples (Fig. 8).

When considering lead ions, the data obtained fit the Freundlich isotherm model better, with R^2^ values ranging from 0.87 to 0.89. This suggests that lead adsorption occurs on a heterogeneous surface. The high Freundlich exponent values of n = 2.4 to 2.75 indicate a favourable adsorption process, dominated by a physicochemical mechanism likely involving ion exchange and the complexation of lead with functional groups present on the biochar surface. The highest sorption capacity (Q_m_) of 0.376 mg/g was recorded for MC_char_chem, while WS_char_chem exhibited the highest Langmuir constant (K_L_ = 90.64 L/mg), indicating a strong binding affinity of lead ions to the adsorbent surface, despite its slightly lower capacity (Table 4). In the case of phenol, the data showed an excellent fit to the Langmuir model, particularly for the WS_char_chem sample, which had an R^2^ of 0.983. This suggests that monolayer adsorption on homogeneous active sites was predominant. This conclusion is further supported by the high values of n in the Freundlich model, which exceeded 4, indicating strong and favourable adsorption. WS_char_chem also demonstrated the highest sorption capacity for phenol (Q_m_ = 0.238 mg/g), confirming its efficiency in removing phenol from aqueous solutions. For MC_char_chem, lower Q_m_ and K_L_ values were observed; however, this cannot be attributed to pore structure limitations, as the biochar exhibited favourable BET parameters and a mean pore diameter within the mesopore range. The most likely reason for the lower values is a reduced number of surface functional groups available for interaction with phenol.

**Table 4:** Parameters of the Freundlich, Langmuir equations, and correlation coefficients for the adsorption of lead and phenol on the studied biochars.

Adsorbent	Langmuir			Freundlich		
	Q_m_, mg/g	K_L_, L/mg	R^2^	K_F,_ L/mg	n	R^2^
Lead						
RC_char_chem	0.298	30.92	0.81	1.562	2.41	0.89
MC_char_chem	0.376	4.14	0.91	1.407	2.46	0.87
WS_char_chem	0.288	90.64	0.71	1.463	2.75	0.87
Phenol						
RC_char_chem	0.117	5.572	0.86	0.117	5.57	0.86
MC_char_chem	0.071	0.035	0.94	0.071	0.04	0.94
WS_char_chem	0.238	4.356	0.983	0.238	4.36	0.98

Summarizing these investigations it can highlighted that biochar has the potential for environmental remediation, particularly in water purification applications. The production and use of biochar, especially that derived from walnut shells through chemical activation, can significantly contribute to achieving certain Sustainable Development Goals (SDGs). Specifically, biochar has proven to be highly effective in removing both organic and inorganic pollutants from water, directly supporting SDG 6: Clean Water and Sanitation. By improving water quality through the application of biochar, we can reduce health risks associated with the presence of heavy metals and other toxins in aquatic ecosystems. Furthermore, producing biochar from agricultural waste and biomass aligns with SDG 12—Responsible Consumption and Production. This practice promotes circular economy principles and decreases the amount of organic waste sent to landfills.

### Conclusions

This study demonstrates the effective valorisation of food industry waste such as rapeseed cake, maize cobs, and walnut shells into high-performance biochar through pyrolysis and further activation processes. The findings highlight the following key points:

1. Char yield ranged from 22–26%, with carbon content exceeding 87% in walnut shell biochar.
2. Chemical activation (using H_3_PO_4_ + ZnCl_2_) significantly enhanced performance, producing specific surface areas up to 557 m^2^/g (WS_char_chem).
3. The contact angle exceeded 90° for chemically activated MC and WS biochars, confirming hydrophobicity favorable for removing organic pollutants.
4. R_50_ values increased after activation, confirming improved thermal stability.
5. The adsorption of lead was effective on a heterogeneous surface and was best described by the Freundlich isotherm model. In contrast, phenol adsorption displayed a monolayer character and fit the Langmuir model very well. The adsorption capacity for lead reached 0.376 mg/g (MC_char_chem), while phenol adsorption peaked at 0.238 mg/g (WS_char_chem). Additionally, 100% of Pb^2+^ and 82% of phenol were removed in batch experiments.
6. The biochars showed no acute toxicity, with Lemna minor growth inhibition below 25%, indicating their environmental safety.

## Electronic supplementary material

Below is the link to the electronic supplementary material.

Supplementary Material 1

## Data Availability

The datasets generated and analysed during the current study are available in the RODBUK Cracow Open Research Data Repository: 10.58032/AGH/YJWHI8.
