# Supplementary material for: Valorisation of food industry waste into high-performance biochar for environmental applications
Source: Sci Rep. 2025 Jul 19;15:26195. doi: 10.1038/s41598-025-11580-z (PMC12274498; doi:10.1038/s41598-025-11580-z)
Supplement: Supplementary file 1 — Supplementary Material 1 [file 41598_2025_11580_MOESM1_ESM.docx]

**Supplementary materials**

**Valorisation of food industry waste into high-performance biochar for environmental applications**

Małgorzata Sieradzka^1^, Wojciech Jerzak^1^, Agata Mlonka-Mędrala^1^, Anna Marszałek^2^, Mariusz Dudziak^2^, Izabela Kalemba-Rec^1^, Aleksandra Błoniarz^1^, Markus Reinmöller^3^, Agnieszka Kopia^1^, Wojciech Nowak^1^, Aneta Magdziarz^1*^

*corresponding author: amagdzia@agh.edu.pl

^1^AGH University of Krakow, Al. Mickiewicza 30, 30-059 Krakow, Poland

^2^Silesian University of Technology, Konarskiego St. 18, 44-100, Gliwice, Poland

^3^Universität Stuttgart, Pfaffenwaldring 23, 70569, Stuttgart, Germany

**Table S1**. Toxicity classification of aqueous solution samples (according to ISO 20079:2006).

| Toxic effect, % | Toxicity class |
| --- | --- |
| ≤ 25.00 | No toxicity |
| 25.01 – 50.00 | Low toxicity |
| 50.01 – 75.00 | Toxicity |
| < 75.00 | High toxicity |

**Table S2.** Fibre analysis of the studied biomass waste

| Fibre | RC | MC | WS |
| --- | --- | --- | --- |
| Hemicellulose (HL), wt.% | 4.16 | 10.80 | 7.05 |
| Cellulose (CL), wt.% | 34.31 | 45.32 | 4.14 |
| Lignin (L), wt.% | 14.18 | 36.33 | 37.57 |

**Table S3.** Chemical and phase composition of biomass ash

| Component | RC | MC | WS |
| --- | --- | --- | --- |
| Na_2_O, wt.% | 0.10 | 0.76 | 0.29 |
| MgO, wt.% | 10.55 | 3.12 | 3.04 |
| Al_2_O_3_, wt.% | 0.22 | 0.34 | 0.44 |
| SiO_2_, wt.% | 1.10 | 5.13 | 1.68 |
| P_2_O_5_, wt.% | 39.86 | 5.85 | 4.90 |
| SO_3_, wt.% | 6.45 | 3.39 | 1.32 |
| Cl, wt.% | 0.27 | 6.34 | 0.29 |
| K_2_O, wt.% | 25.08 | 71.50 | 51.72 |
| CaO, wt.% | 15.74 | 2.42 | 24.57 |
| Fe_2_O_3_, wt.% | 0.37 | 0.41 | 0.29 |
| NiO, wt.% | 0.02 | 0.04 | 0.02 |
| CuO, wt.% | 0.02 | 0.06 | 0.28 |
| ZnO, wt.% | 0.14 | 0.13 | 0.00 |
| SnO_2_, wt.% | - | - | 3.60 |
| PbO, wt.% | - | - | 7.38 |
| *Phase composition* |  |  |  |
|  | K_2_CaP_2_O_7_ | KHCO_3_ | K_2_Ca(CO_3_)_2_ |
|  | CaHPO_4_, CaCO_3_  K_2_SO_4_ | KCl, SiO_2_ | KHCO_3_  Ca_2_PO_4_, SiO_2_, PbO |

| 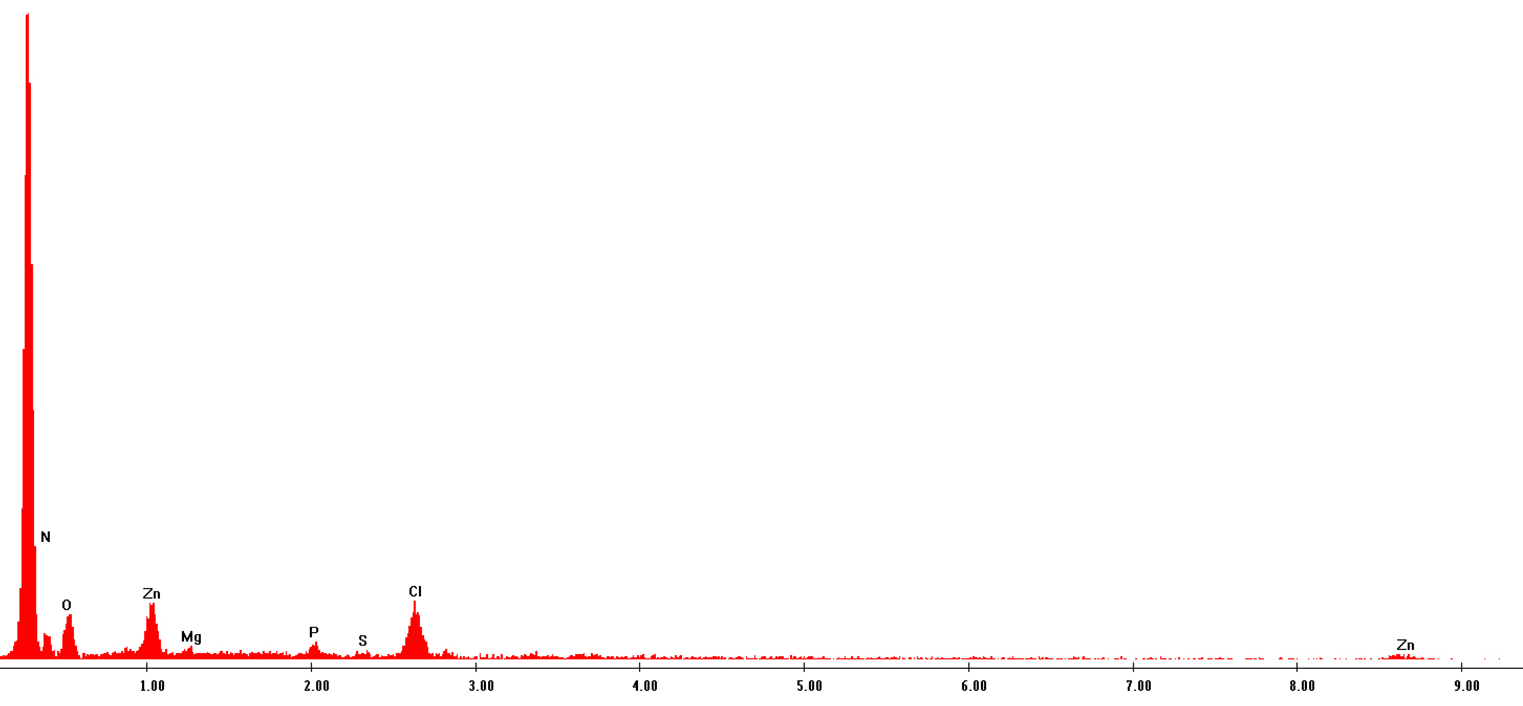 |
| --- |

**Figure 1S.** A representative of EDS spectrum for rape cake.

**Figure 2S.** DTG of biochars from a) rape cake, b) maize cob and c) walnut shells and biochars activated physically (“phys”) and chemically (“chem”) compared to graphite.
